# Connectivity Mapping Using a Novel *sv2a* Loss-of-Function Zebrafish Epilepsy Model as a Powerful Strategy for Anti-epileptic Drug Discovery

**DOI:** 10.3389/fnmol.2022.881933

**Published:** 2022-05-24

**Authors:** Yifan Zhang, Lise Heylen, Michèle Partoens, James D. Mills, Rafal M. Kaminski, Patrice Godard, Michel Gillard, Peter A. M. de Witte, Aleksandra Siekierska

**Affiliations:** ^1^Laboratory for Molecular Biodiscovery, KU Leuven, Leuven, Belgium; ^2^Department of Neuropathology, Amsterdam Neuroscience, Amsterdam UMC, University of Amsterdam, Amsterdam, Netherlands; ^3^Department of Clinical and Experimental Epilepsy, UCL Queen Square Institute of Neurology, London, United Kingdom; ^4^Chalfont Centre for Epilepsy, Chalfont St Peter, United Kingdom; ^5^Department of Medicinal Chemistry, Faculty of Pharmacy, Jagiellonian University Medical College, Kraków, Poland; ^6^UCB Pharma, Braine-l’Alleud, Belgium

**Keywords:** zebrafish, SV2A, levetiracetam, epilepsy, connectivity mapping (CMap)

## Abstract

Synaptic vesicle glycoprotein 2A (SV2A) regulates action potential-dependent neurotransmitter release and is commonly known as the primary binding site of an approved anti-epileptic drug, levetiracetam. Although several rodent knockout models have demonstrated the importance of SV2A for functional neurotransmission, its precise physiological function and role in epilepsy pathophysiology remains to be elucidated. Here, we present a novel *sv2a* knockout model in zebrafish, a vertebrate with complementary advantages to rodents. We demonstrated that 6 days post fertilization homozygous *sv2a^–/–^* mutant zebrafish larvae, but not *sv2a*^+/–^ and *sv2a^+/+^* larvae, displayed locomotor hyperactivity and spontaneous epileptiform discharges, however, no major brain malformations could be observed. A partial rescue of this epileptiform brain activity could be observed after treatment with two commonly used anti-epileptic drugs, valproic acid and, surprisingly, levetiracetam. This observation indicated that additional targets, besides Sv2a, maybe are involved in the protective effects of levetiracetam against epileptic seizures. Furthermore, a transcriptome analysis provided insights into the neuropathological processes underlying the observed epileptic phenotype. While gene expression profiling revealed only one differentially expressed gene (DEG) between wildtype and *sv2a*^+/–^ larvae, there were 4386 and 3535 DEGs between wildtype and *sv2a^–/–^*, and *sv2a*^+/–^ and *sv2a^–/–^* larvae, respectively. Pathway and gene ontology (GO) enrichment analysis between wildtype and *sv2a^–/–^* larvae revealed several pathways and GO terms enriched amongst up- and down-regulated genes, including MAPK signaling, synaptic vesicle cycle, and extracellular matrix organization, all known to be involved in epileptogenesis and epilepsy. Importantly, we used the Connectivity map database to identify compounds with opposing gene signatures compared to the one observed in *sv2a^–/–^* larvae, to finally rescue the epileptic phenotype. Two out of three selected compounds rescued electrographic discharges in *sv2a^–/–^* larvae, while negative controls did not. Taken together, our results demonstrate that *sv2a* deficiency leads to increased seizure vulnerability and provide valuable insight into the functional importance of *sv2a* in the brain in general. Furthermore, we provided evidence that the concept of connectivity mapping represents an attractive and powerful approach in the discovery of novel compounds against epilepsy.

## Introduction

Epilepsy, affecting individuals of all ages regardless of gender or socio-economic status, is a chronic brain disease characterized by an ongoing liability to unprovoked recurrent epileptic seizures due to excessive, hypersynchronous discharges of neurons in the brain (Stafstrom and Carmant, 2015). Epilepsy can be a result of an insult to the brain such as injury, stroke, central nervous system (CNS) infections, and vascular or congenital brain malformations (Engel, 2001; Singh and Trevick, 2016), or can be a consequence of a genetic variant in one or more proteins that control brain excitability. Although methods such as surgery, vagus nerve stimulation, and dietary changes have been employed to treat epilepsy, anti-epileptic drugs (AEDs) are the most commonly used treatment approach. Current marketed AEDs consist of a variety of structural classes acting through a range of different mechanisms, for instance by modulation of voltage-gated ion channels, glutamate-mediated excitatory neurotransmission, and GABA-mediated inhibitory pathways (Löscher, 2017). In contrast, levetiracetam primarily exerts its antiseizure activity by selectively binding to synaptic vesicle glycoprotein 2A (SV2A) in the brain (Loscher et al., 2016). Levetiracetam is a first-line AED clinically used for focal, myoclonic, and generalized tonic-clonic seizures. Additionally, it has been used in an off-label manner for the treatment of status epilepticus (SE) and other non-epileptic conditions such as movement disorders and neuropathic pain (Abou-Khalil, 2008; Crepeau and Treiman, 2010; Meehan et al., 2011), as well as it could possibly be effective in patients with Alzheimer’s disease to improve cognitive symptoms (Kong et al., 2021; Vossel et al., 2021).

The SV2 family is well conserved among vertebrates (Chang and Sudhof, 2009) and in mammals consists of SV2A, SV2B, and SV2C (Sills, 2010). All members are integral membrane proteins composed of a highly N-glycosylated 80 kDa backbone comprised of 12 transmembrane (TM) helices organized in 2 TM domains, two large loops (cytoplasmic and intravesicular) differing among the isoforms, and N- and C-terminal cytoplasmic sequences (Bartholome et al., 2017). Multiple N-glycosylation sites appear to be essential for proper folding and correct trafficking of SV2 in the synapses (Scott and Panin, 2014). SV2 isoforms are not neurotransmitter-specific and have varying distribution in the brain (Bartholome et al., 2017) with SV2A being the most abundant and ubiquitously expressed in almost all the neurons, and also found in neuroendocrine cells and neuromuscular junctions (Crèvecœur et al., 2013). SV2A functions as a synaptic vesicle glycoprotein essential for neurotransmitter release. Specifically, SV2A modulates the readily available synaptic vesicle pool and synaptic vesicle size (Xu and Bajjalieh, 2001). It is also involved in calcium-dependent neurotransmitter exocytosis by regulating the expression and trafficking of a synaptic vesicle calcium sensor protein, synaptogamin (Yao et al., 2010), and contributing to a maturation step of primed vesicles rendering them competent for release (Nowack et al., 2010).

Nevertheless, the precise physiological role of SV2A remains to be elucidated. Studies in animal models demonstrated that *SV2A* loss-of-function caused premature mortality and severe epilepsy. *SV2A* knockout mice appeared normal at birth, but exhibited spontaneous fatal seizures after 1–2 weeks, without any obvious abnormalities in the brain or synapse morphology, followed by death within 3 weeks (Crowder et al., 1999; Janz et al., 1999; Menten-Dedoyart et al., 2016). Partial *SV2A* deficiency in *SV2A* heterozygous mice did not cause spontaneous seizure activity, however, it enhanced drug-induced seizure vulnerability and accelerated epileptogenesis (Kaminski et al., 2009). Similarly, homozygous rats carrying an *SV2A*-targeted missense mutation exhibited normal development but were susceptible to PTZ induced-seizures and kindling development (Tokudome et al., 2016). Additionally, a mutation in *SV2A* caused photosensitive reflex epilepsy in the Fepi chicken model (Douaud et al., 2011). Furthermore, electrophysiological studies on hippocampal slices from wildtype and *SV2A* knockout mice indicated that deletion of *SV2A* had more of a suppressive effect on the frequency and amplitude of inhibitory postsynaptic currents (IPSC) than on excitatory currents, likely resulting in seizure onset and epilepsy (Bartholome et al., 2017). A decreased *SV2A* expression was also found in rodent models of temporal lobe epilepsy (TLE), further supporting the hypothesis that *SV2A* deficiency may contribute to epileptogenic progression and seizure initiation (Gorter et al., 2006; De Smedt et al., 2007). Dysfunction of SV2A impaired the synaptic GABA release by reducing the synaptogamin 1 levels, highlighting the imbalance between excitatory and inhibitory neurotransmission in modulating the epileptogenic processes (Crowder et al., 1999; Tokudome et al., 2016).

In humans, only three variants have been reported to link *SV2A* to epilepsy. A homozygous R383Q missense mutation in *SV2A* gene resulted in infantile-onset intractable epilepsy, involuntary movements, microcephaly, and developmental delay (Serajee and Huq, 2015). Moreover, recently also rare heterozygous variants (R570C and G660R) were found in two infantile patients with drug-resistant epilepsy with myoclonic and generalized clonic-tonic seizures (Wang et al., 2019; Calame et al., 2021). Treatment with levetiracetam did not alleviate the patients’ seizures or surprisingly even worsened the epileptic symptoms until treatment discontinuation (Wang et al., 2019; Calame et al., 2021). Furthermore, clinical studies investigated the importance of SV2A in normal brain functioning and its pathogenic potential in the case of genetic disorders (Bartholome et al., 2017). For instance, reduced *SV2A* expression was observed in epileptic foci resected from patients with drug-resistant TLE (Feng et al., 2009), TLE with hippocampal sclerosis (van Vliet et al., 2009), focal cortical dysplasia, and tuberous sclerosis complex (Toering et al., 2009).

Although traditional drug discovery strategies, including either molecular (target-based) or empirical (phenotypical) approaches, have led to the development of about 30 AEDs with diverse molecular targets, there are still many challenges in the pharmacological treatment of epilepsy. Recent advances in network-oriented approaches and “omics” technologies have led to a range of novel computational strategies being orthogonal to current concepts of drug discovery (Musa et al., 2018). One of these emerging approaches is called ‘connectivity mapping’ whereby potential compounds are judged not by their binding affinity to a particular target or reversion of a certain phenotype, but by their ability to induce a transcriptional response opposite to the one underpinning the disease state (Lamb et al., 2006). For this, large reference perturbation databases (e.g., CMap and LINCS) containing transcriptome profiles of dozens of cultivated cell lines treated with thousands of chemical compounds were created, that can be queried by researchers worldwide. Since the first introduction of the concept and CMap database in 2006, several successful applications have been reported in distinct disease areas (Ishimatsu-Tsuji et al., 2010; Li H. et al., 2020; Scott et al., 2020). For instance, recently connectivity mapping identified metformin, nifedipine, and pyrantel tartrate as candidate anti-epileptics, which was confirmed in a larval zebrafish seizure model (Brueggeman et al., 2019).

Zebrafish are lower vertebrates that possess many advantages over classical rodent models including high genetic and physiological homology to humans, high fecundity, external fertilization, transparency through early larval stages that enables powerful imaging techniques, and ease of various genetic manipulations (Hortopan et al., 2010; Copmans et al., 2017). Due to these attributes, they have successfully been deployed in large-scale, systematic drug discovery screens to identify small molecules that can suppress disease phenotypes (MacRae and Peterson, 2015; Rennekamp and Peterson, 2015; Patton et al., 2021). Importantly, zebrafish are an established model for studying epilepsy disorders (Copmans et al., 2017; Yaksi et al., 2021). The development of mutant zebrafish models in recent translational research has allowed rapid functional determination of candidate epilepsy genes (Baraban et al., 2013; Samarut et al., 2018; Swaminathan et al., 2018; Siekierska et al., 2019).

In the current study, we explored the role of SV2A in the brain development and pathophysiology of epilepsy using an *in vivo* zebrafish model. To that end, we disrupted the *sv2a* function in zebrafish by generating a knockout line using CRISPR/Cas9 technology. Our results demonstrated that *sv2a* deficiency leads to premature death, hyperactivity, and severe spontaneous epileptiform discharges. Pharmacological validation of the *sv2a* model demonstrated a partial rescue of the epileptiform discharges after treatment with levetiracetam, indicating that other mechanisms, in addition to Sv2a, are likely to be involved. Moreover, transcriptome analysis identified dysregulated genes in several pathways essential for correct brain development and involved in epileptogenesis. Furthermore, we successfully applied connectivity mapping to identify compounds that reverted the epileptic phenotype of *sv2a^–/–^* larvae. Altogether, our results provide causative factors for *SV2A* loss-of-function epileptogenesis and indicate promising alternative directions in the discovery of AEDs.

## Materials and Methods

### Zebrafish Husbandry

Adult zebrafish (*Danio rerio*) were maintained in a UV-sterilized recirculating system equipped with a mechanical and biological filtration unit and kept under a 14/10 h light/dark cycle at the temperature of 27–28°C and a pH of 6.8–7.5. Water quality was monitored for pH, temperature, conductivity, ammonia, nitrite (SL1000 Portable Parallel Analyzer, Hach Instruments, United States), and nitrate levels (Tetra, Melle, Germany). Zebrafish were fed three times per day with flake food (TetraMin, Tetra, Germany) and Artemia (brine shrimp). Embryos were obtained via natural spawning and were kept in petri dishes (92 × 16 mm, Sarstedt, Nümbrecht, Germany) at 28°C in a Peltier-cooled incubator (IPP 260, Memmert, Schwabach, Germany) in Danieau’s medium (1.5 mM HEPES, pH 7.2, 17.4 mM NaCl, 0.21 mM KCl, 0.12 mM MgSO_4_, 0.18 mM Ca(NO_3_)_2_ and 0.6 μM methylene blue). All zebrafish experiments were approved by the Ethics Committee of the KU Leuven (P023/2017 and P027/2019) and by the Belgian Federal Department of Public Health, Food Safety, and Environment (LA1210199).

### Establishing *sv2a* Knockout Zebrafish Line Using CRISPR/Cas9 Method

Single-guide RNA (sgRNA) targeting the antisense strand of zebrafish *sv2a* exon 2 (5′-GTGGACCCTCTACTTTGTGC(GGT)-3′) was designed and synthesized by GeneArt^®^ (Thermo Fisher Scientific). *sv2a* sgRNA mRNA was transcribed using MegaScript T7 Kit (Ambion), DNase treated (TURBO DNase, Life Technologies) and was purified with MEGAclear Transcription Clean-Up Kit (Ambion). Cas9 nuclease mRNA was purchased from GeneArt (Invitrogen). Next, a mix of 150 pg Cas9 mRNA and 7 pg *sv2a* sgRNA in 1 nl volume was co-injected into early one-cell stage wildtype embryos of AB line using a Femtojet 4i pressure microinjector (Eppendorf) and a M3301R Manual Micromanipulator (WPI). The injected embryos were raised till adulthood. To identify founders with germline transmitted mutation and high rate of indel mutations, each of the F0 CRISPR/Cas9-injected adults was outcrossed with a wildtype. F1 offspring was collected, individually lysed to obtain genomic DNA for subsequent PCR amplification with primers flanking the potential mutated target site, screened by Sanger sequencing (LGC Genomics, Germany), and analyzed with SeqMan Pro software (DNASTAR, Lasergene). F1 generation embryos of the selected F0 founder were raised to adulthood, fin clipped and Sanger sequenced. Individuals carrying the same mutation (1 bp insertion of G at CRISPR site) were identified and pooled together. All experiments were performed on F2 generation adults.

### Genotyping of the Larvae Using High Resolution Melting Analysis

To extract genomic DNA, a fin clip of a larva was placed in a separate tube with 20 μl of 50 mM NaOH and heated at 95°C for 10 min, followed by neutralization with 100 mM Tris HCl (pH 8.0) (1/10 volume). Lysed samples were genotyped by performing a PCR reaction with Precision Melt Supermix for high resolution melting (HRM) analysis (Bio-Rad #172-5112) and *sv2a*-specific primers (Supplementary Table 1) in a CFX96 touch RT-PCR detection system (Bio-Rad) using Hardshell^®^ Low Profile Thin-wall 96-well skirted PCR plates (Bio-Rad). Curves were analyzed using the Precision Melt Analysis™ Software (Bio-Rad). Genotypes of the individual larvae clustering together were confirmed by Sanger sequencing (LGC Genomics, Germany) and were analyzed using SeqMan software (DNASTAR, Lasergene).

### Survival Assay and Morphology

Larvae were cultured in Danieau’s medium and scored daily up to 10 days post fertilization (dpf) for any morphological or behavioral abnormalities (e.g., pericardial edema, head, eye, or jaw malformations, bent spine, and lack of touch response) and lethality. The larvae were photographed using a stereomicroscope (Leica MZ10 F) equipped with a digital camera (DFC310 FX) and Leica Application Suite software (version 3.6.0). Dead larvae were immediately collected for genotyping as described before.

### Locomotor Activity Assessment

6 dpf zebrafish larvae were transferred individually into a 96-well plate containing 100 μl Danieau’s medium per well. After positioning the plate inside a DanioVision box (Noldus, Netherlands), larval behavior was recorded for 10 min in the light after 30 min habituation period in the dark. Ethovision XT16 (Noldus) was used to quantify locomotor behavior as total velocity (mm/s).

### Non-invasive Local Field Potential Recordings

Electrographic brain activity of 5 and 6 dpf larvae was assessed by non-invasive local field potential (LFP) recordings from the optic tectum as described previously (Heylen et al., 2021). A blunt glass electrode (soda lime glass, Hilgenberg, Germany) pulled with DMZ Universal Puller (Zeitz, Germany) to an opening of approximately 15–20 microns, connected to a high-impedance amplifier, was filled with artificial cerebrospinal fluid (124 mM NaCl, 2 mM KCl, 2 mM MgSO_4_, 2 mM CaCl_2_, 1.25 mM KH_2_PO_4_, 26 mM NaHCO_3_ and 10 mM glucose) and positioned on the skin above the optic tectum of a larva embedded in 2% low-melting point agarose (Thermo Fisher Scientific). The differential signal between the recording electrode and the reference electrode was amplified 10,000 times by EXT-02F/2 extracellular amplifier (NPI Electronic), band pass filtered at 3–300 Hz and digitized at 2 kHz via a PCI-6251 interface (National Instruments) with WinEDR (John Dempster, University of Strathclyde). A HumBug noise eliminator (Quest Scientific) was used to remove 50–60 Hz noise. Spontaneous discharges were considered as epileptiform events when their amplitudes exceeded three times the baseline and lasted for at least 100 ms. Each recording lasted for 10 min and was visualized with Clampfit 10.2 software (Molecular Devices Corporation). In order to quantify the signal from epileptiform brain discharges, we employed a specifically developed software (Li J. et al., 2020). In brief, LFP recordings were examined by Welch’s power spectral density (PSD) analysis, using 100 ms long windows extracted with a Hamming window and 80% overlap. The window length was chosen considering that an epileptiform discharge may last as short as 50–100 ms. Subsequently, the average spectral power in consecutive 10 Hz frequency bands (i.e., 0–10 Hz, 10–20 Hz, 140–150 Hz) was computed. The magnitude of the average PSD values gives an indication about the number of epileptiform discharges throughout the recordings in each frequency band.

### Pharmacological Evaluation

Sodium valproate, levetiracetam, CAY-10415, digoxin, and calmidazolium HCl were purchased from Sigma. Alvocidib, AS-605240, and PD-98059 were purchased from Selleckchem. All compounds were dissolved in dimethyl sulfoxide (DMSO) and kept at −20°C. For experiments, stock solutions were diluted in Danieau’s medium to achieve a final DMSO concentration of 1% v/v. As a vehicle (VHC) control 1% DMSO in Danieau’s medium was used. Before performing pharmacological experiments, maximum tolerated concentration (MTC) of the compounds was determined as described previously (Zhang et al., 2017): sodium valproate 500 μM, levetiracetam 7 mM, alvocidib 2 μM, PD-98059 12 μM, AS-605240 800 nM, CAY-10415 8 μM, digoxin 4 μM, and calmidazolium HCl 1 μM. In brief, 12 larvae of 5 dpf were transferred individually into a 96-well plate containing 100 μl volume per well and exposed to a certain concentration (twofold dilution series, starting from the highest soluble concentration). After 22-h exposure, the following parameters were investigated: touch response, morphology, posture, edema, signs of necrosis, presence of swim bladder and heartbeat. The MTC was defined as the highest soluble concentration at which no larvae died nor showed signs of toxicity or locomotor impairment in comparison to VHC-treated control larvae. For pharmacological evaluation, larvae were treated with VHC or compounds at their respective MTC 22 h prior to an experiment.

### Hematoxylin and Eosin Staining

Hematoxylin and eosin staining was performed on 6 dpf larvae as described previously (Siekierska et al., 2019; Partoens et al., 2021). Larvae were anesthetized in 0.008% tricaine and fin clipped for genotyping. Subsequently, they were fixed in 4% paraformaldehyde (PFA) and kept in 70% ethanol. At least four larvae per genotype were embedded in 1% agarose (Invitrogen) in 1x TAE buffer (Thermo Fisher Scientific). A customized mold was utilized to align zebrafish larvae and produce agarose blocks with identically distributed wells of the same depth. Agarose blocks were put in an enclosed automated tissue processor (Shandon Excelsior ES, Thermo Fisher Scientific) for overnight dehydration and embedded in paraffin. Larval heads were sectioned using a HM 325 manual rotary microtome (Thermo Fisher Scientific) at a thickness of 5 μm and sections were applied to microscope slides. Slides were stained with hematoxylin and eosin using Varistain™ Gemini ES Automated Slide Stainer (Thermo Fisher Scientific) according to laboratory protocols. The resulting sections were imaged at 20× and 40× magnification in SPOT 5.1 software (SPOT Imaging) by a SPOT-RT3 camera mounted on a Leica microscope. Four equivalent sections were selected for each genotype, and hematoxylin-positive stained nuclei were counted using QuPath (0.1.2) software to support size difference. The results were expressed as average hematoxylin-positive stained nuclei within a selected brain area.

### RNA Sequencing

For RNA sequencing, 6 dpf zebrafish larvae were anesthetized and decapitated using a razor blade. 10 heads were pooled in quadruplicate per genotype and RNA was extracted as described below. RNA concentration and purity were determined spectrophotometrically using the NanoDrop ND-1000 (NanoDrop Technologies) and RNA integrity was assessed using a Bioanalyser 2100 (Agilent). A total of 500 ng of total RNA per sample was used as input for sample preparation. Library preparation and sequencing were completed by VIB nucleomics core.^1^ TruSeq RNA-Seq sample preparation kit was used to prepare sequencing library of mRNA in accordance with the manufacturer guidelines. This library was subjected to single-end sequencing on a NextSeq500 v2 High75 flow-cell. Sequencing data are available in the ArrayExpress database^2^ under accession number E-MTAB-11505.

### Bioinformatic Analysis

Read quality was assessed using ShortRead 1.36.1 package from Bioconductor^3^ (Morgan et al., 2009). Low quality ends (<Q20) and adapters (at least 10 nt overlap and 90% match) were trimmed using FastX 0.0.14 (Hannon, 2010) and cutadapt 1.15 (Martin, 2011), respectively. If any of the reads dropped below 35 nt in length they were excluded. Poly-A reads (more than 90% of the bases equaling A), ambiguous reads and low quality reads (more than 50% of the bases < Q25) were removed with FastX 0.0.14 and ShortRead 1.36.1. Using bowtie 2.3.3.1 (Langmead and Salzberg, 2012), reads that aligned to phix illumina were identified and removed. Reads were aligned to the zebrafish reference genome, GRCz11, using STAR 2.5.2b (Dobin et al., 2013). The aligned reads were then passed to samtools 1.5 (Li et al., 2009) for quality filtering, sorting and indexing. Quantification of expression levels was performed with featureCounts 1.5.3 (Liao et al., 2014). The unnormalized RNA count matrix was passed on to the R package DESeq2 (Love et al., 2014). Genes were considered expressed if it had 1 counts-per-million in at least one condition. Gene expression changes with a Benjamini-Hochberg adjusted *p*-value < 0.05 were considered statistically significant.

### Gene Ontology and Pathway Enrichment Analysis

The Gene Ontology (GO) knowledgebase (Ashburner et al., 2000) and Kyoto Encyclopedia of Genes and Genome (KEGG) (Kanehisa and Goto, 2000) for zebrafish were queried to test DEGs for gene ontology and pathway enrichment, respectively, using the Enrichr package (Chen et al., 2013). Enriched GO term lists containing the top 10 terms amongst molecular function, cellular component and biological process were processed using the ‘Enrichment Map’ plugin for Cytoscape^4^ to create a visual representation of the GO enrichment (Isserlin et al., 2014).

### RNA Extraction and RT-qPCR Analysis

Total RNA was extracted using TRIzol reagent (Invitrogen), followed by phenol-chlorophorm extraction and isopropanol precipitation. After ethanol washes, the RNA pellet was air-dried, dissolved in nuclease-free water (Fermentas) and treated with RNase-free DNase (Roche) to remove possible genomic DNA contamination. Reverse transcription of 2 μg of total RNA to single-stranded cDNA was performed using random primers and SuperScript III Reverse Transcriptase (Invitrogen) according to the manufacturer’s instructions. Next, the generated cDNA was diluted (1:20) and amplified using gene specific primers (Supplementary Table 1) and 2x SsoAdvanced Universal SYBR Green Supermix (Bio-Rad) in HardShell^®^ Low-Profile Thin-Wall 96-Well Skirted PCR Plates (Bio-Rad) on CFX96 Touch Real-Time PCR Detection System (Bio-Rad) under cycling conditions according to the manufacturer’s protocol. The relative expression levels were quantified using the comparative Cq method (ΔΔCq) with CFX Maestro software (Bio-Rad). Transcripts were normalized against 18s housekeeping gene that was experimentally determined to have the most stable expression in our reaction conditions.

### Connectivity Map Analysis

Connectivity map (CMap) uses cellular responses to genetic and chemical perturbations to connect diseases with genes underlying them and therapeutics to treat them (Lamb et al., 2006). After converting zebrafish identifiers to their human orthologs using BioMart (Durinck et al., 2009), the top 50 up- and top 50 down-regulated genes in *sv2a^–/–^* zebrafish sorted by Wald statistic were fed into the CMap query application. These were then compared to the CMap library containing over one and a half million gene expression signatures derived from the L1000 platform, a high-throughput gene expression assay, to identify compounds reversing the entered disease expression profile. Each compound was assigned a connectivity score between −100 and 100 for the supplied list: while a positive score indicates a similarity between a given compound signature and that of the query, a negative score indicates that the two signatures are opposing. Compounds targeting master regulators of cellular homeostasis and proliferation, such as “DNA synthesis,” “RNA synthesis,” “tRNA synthetase,” “RNA polymerase,” and “protein synthesis,” were not considered for further investigation. Based on availability, three compounds with opposing gene signatures (scores < −90), together with three compounds with highly similar or non-matching signatures (scores > 90 or equaling 0, negative controls) were selected for further testing, as described previously (see Pharmacological evaluation).

### Statistical Analysis

Unpaired Student’s *t*-test was used to compare means between two groups; for three or more groups one-way or two-way ANOVA followed by Dunnett’s or Sidak’s multiple comparison test, respectively, was used. Log-rank (Mantel-Cox) test was used for Kaplan-Meier curves analysis. Outliers were identified using the ROUT method (*Q* = 1%). Statistical analyses were performed using GraphPad Prism 9. Significance of all statistical comparisons was set at *p* < 0.05.

## Results

### CRISPR/Cas9-Mediated Generation of *sv2a* Loss-of-Function in Zebrafish

**FIGURE 1:**
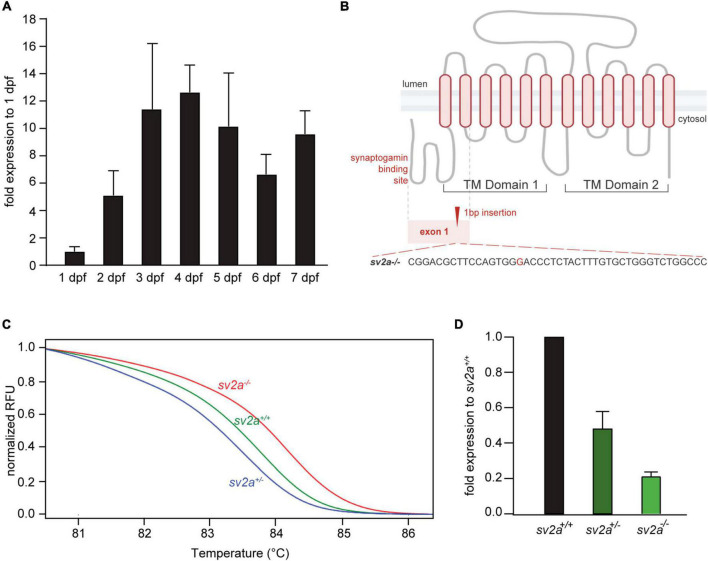
*sv2a* expression in developing zebrafish larvae (1-7 dpf) and CRISPR-mediated *sv2a* knockout in zebrafish. **(A)** qPCR analysis of *sv2a* mRNA levels in wildtype larvae normalized to 18s and represented as the fold expression to 1 dpf. Values are reported as mean ± SD of three independent experiments. **(B)** Schematic representation of the SV2A protein with the two distinct transmembrane (TM) domains and the cytosolic synaptogamin binding site. Gray dashed line indicates exon 1 encoding synaptogamin binding domain and the first two TM helices. Red dashed line magnifies the region with the 1 nucleotide insertion (G) at the target site in exon 1. **(C)** High resolution melting (HRM) curve analysis discriminates *sv2a^+/+^*, *sv2a*^+/–^, and *sv2a^–/–^* zebrafish larvae at 6 dpf. **(D)** qPCR analysis of *sv2a* mRNA levels in *sv2a^+/+^*, *sv2a*^+/–^, and *sv2a^–/–^* larvae at 6 dpf normalized to 18s and represented as the fold expression to *sv2a^+/+^* larvae. Values are reported as the mean ± SD of three independent experiments.

Zebrafish *sv2a* gene is the only ortholog of the human *SV2A* gene. It displays 79.6% overall amino acid identity with the human protein and all important ligand (levetiracetam) binding residues are conserved (Supplementary Figure 1A; Lee et al., 2015). qPCR analysis of *sv2a* expression during 1-7 dpf in wildtype zebrafish larvae revealed that *sv2a* was already detectable at first day of embryonic development and its expression gradually increased reaching a plateau at 3-4 dpf (Figure 1A). In addition, the affinity of levetiracetam for the zebrafish Sv2a protein was evaluated *in vitro* and is in line with values reported for recombinant and native human and rat SV2A (Supplementary Figure 1B; Gillard et al., 2006; Gillard et al., 2011; Wood et al., 2020). Therefore, zebrafish is a relevant *in vivo* model to investigate the function of SV2A. To generate a knockout zebrafish model with loss of *sv2a* function, we targeted early exon 1 of the *sv2a* transcript (ENSDART00000172232.2), located in the synaptogamin-binding domain (Figure 1B). We simultaneously injected *sv2a*-specific sgRNA targeting exon 2 and Cas9 mRNA into single-cell zebrafish embryos. We selected a positive founder carrying a 1-bp (G) insertion, which results in a frameshift of the *sv2a* transcript leading to a premature stop codon at position 186 (Figure 1B). We confirmed the mutated genomic sequence by Sanger sequencing and HRM assay (Figure 1C). The selected founder was outcrossed with a wildtype adult zebrafish and F1 generation offspring bearing the frameshift mutation was pulled together to generate F2 offspring, which was used to perform the experiments. Via RT-qPCR, we confirmed that there was a reduction of *sv2a* mRNA by 80% in *sv2a^–/–^* larvae at 6 dpf and approximately half of the transcript present in *sv2a*^+/–^ larvae (Figure 1D), proving the efficiency of the knockout likely leading to a full loss of Sv2a function in homozygous zebrafish.

### *sv2a* Loss-of-Function Leads to Early Lethality in Zebrafish Embryos

**FIGURE 2:**
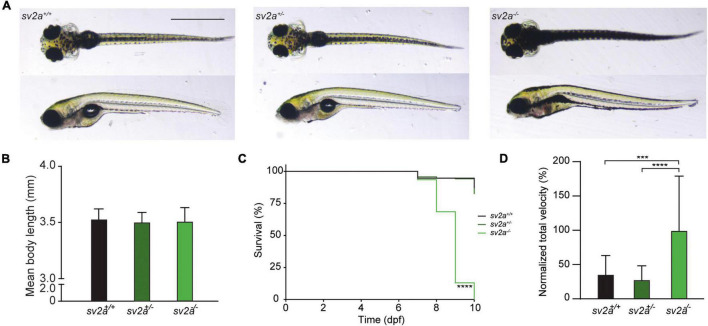
Morphological, survival and behavioral analysis of *sv2a* knockout zebrafish. **(A)** Representative images of sv*2a*^+/+^, *sv2a*^+/–^, and *sv2a ^–/–^* zebrafish larvae at 6 dpf. Scale bar 1 mm. **(B)** Comparison of mean body length of *sv2a*^+/+^ (*n* = 8), *sv2a*^+/–^ (*n* = 8), and *sv2a ^–/–^* (*n* = 8) zebrafish larvae at 6 dpf (mean ± SD). **(C)** Kaplan-Meier curve demonstrating survival of *sv2a*^+/+^ (*n* = 94), *sv2a*^+/–^ (*n* = 208), and *sv2a^–/–^* (*n* = 92) zebrafish larvae. Significant larval death in *sv2a^–/–^* larvae can be observed in a time-dependent manner. Statistical analysis was performed using Log-rank (Mantel-Cox) test (****p < 0.0001). **(D)** Locomotor activity of *sv2a*^+/+^ (*n* = 15), *sv2a*^+/–^ (*n* = 53), and *sv2a^–/–^* (*n* = 40) zebrafish larvae at 6 dpf. Data were assessed over the total tracking period of 10 min, normalized to *sv2a^–/–^* larvae, and expressed as normalized total velocity (%) (mean ± SD). Statistical analysis was performed by one-way ANOVA followed by Dunnett’s multiple comparison (***p < 0.001, ****p < 0.0001). Data were collected from four independent experiments.

Homozygous *sv2a^–/–^* mutant larvae had a normal appearance without major morphological abnormalities. The majority of the larvae at 6 dpf displayed some minor defects such as lack of swim bladder, slight curvature on the body axis, and, interestingly, darker pigmentation (Figure 2A). Heterozygous *sv2a*^+/–^ larvae were morphologically similar to their wildtype siblings (Figure 2A). We also did not find any significant difference in body length between the different genotypes (Figure 2B, data shown at 6 dpf). The survival analysis demonstrated that *sv2a^–/–^* larvae died prematurely between 8 and 10 dpf (Figure 2C), whereas their wildtype and heterozygous siblings could be raised until adulthood. Subsequently, we conducted behavioral tracking to quantify the individual larval movement as total velocity (mm/s). Interestingly, *sv2a^–/–^* larvae were hyperactive in comparison to *sv2a*^+/–^ and *sv2a*^+/+^ larvae (Figure 2D). Of note, other parameters, including total activity (%), total mobility (%) and total distance moved (mm), were assessed as well and showed a similar elevation in *sv2a^–/–^* larvae as observed for total velocity (mm/s) (data not shown).

### *sv2a* Knockout Zebrafish Larvae Display Spontaneous Electrographic Seizures

**FIGURE 3:**
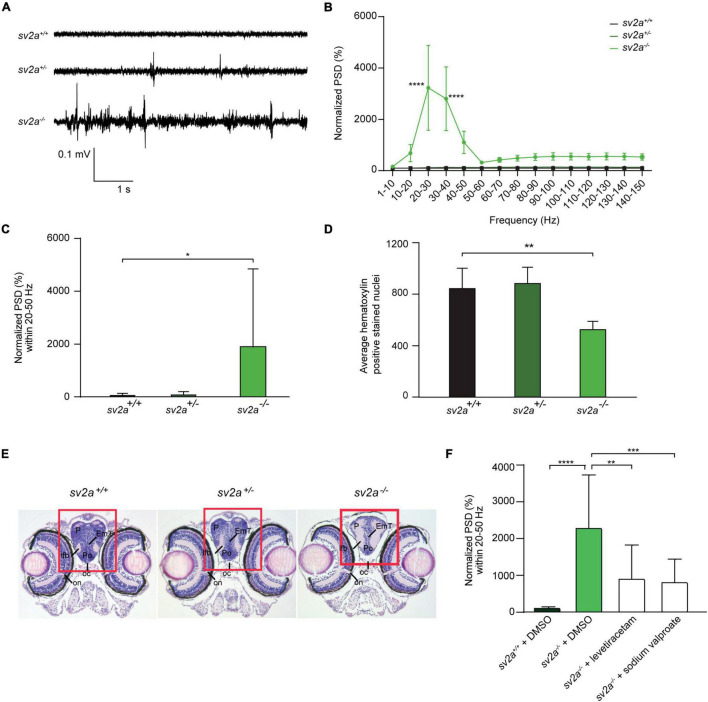
Spontaneous electrographic seizures and brain malformation in *sv2a* knockout zebrafish. **(A)** Representative local field potential (LFP) recordings of *sv2a*^+/+^, *sv2a*^+/–^, and *sv2a ^–/–^* zebrafish larvae at 6 dpf. **(B)** Power spectral density (PSD) analysis of *sv2a*^+/+^ (*n* = 9), *sv2a*^+/–^ (*n* = 11), and *sv2a ^–/–^* (*n* = 10) zebrafish larvae at 6 dpf. A significant increase in PSD values was observed in *sv2a^–/–^* larvae compared to *sv2a*^+/+^ larvae. Results were normalized to *sv2a*^+/+^ larvae as 100%. Statistical analysis was performed using two-way ANOVA with Sidak’s multiple comparisons test (****p < 0.0001 compared to *sv2a*^+/+^). **(C)** PSD values (mean ± SD) plotted per condition over the 20–50 Hz region. Statistical analysis was performed using one-way ANOVA followed by Dunnett’s multiple comparison (*p < 0.05). **(D)** Hematoxylin-positive stained nuclei (mean ± SD) counted using QuPath (0.1.2) software. The region with significant loss of hematoxylin-positive stained nuclei was highlighted in red. The results are expressed as mean ± SD of 4 equivalent sections per genotype. Statistical analysis was performed using one-way ANOVA followed by Dunnett’s multiple comparison (**p < 0.01). **(E)** Hematoxylin and eosin staining of paraffin-embedded coronary sections from the forebrain of *sv2a*^+/+^, *sv2a*^+/–^, and *sv2a ^–/–^* zebrafish larvae at 6 dpf. Scale bar 100 μm. oc, optic chiasm; on, optic nerve; Po, preoptic region; P, pallium; lfb, lateral forebrain bundle. **(F)** Effect of levetiracetam and sodium valproate on locomotor behavior and epileptiform brain activity in 6 dpf *sv2a^–/–^* zebrafish larvae. PSD values (mean ± SD) from LFP recordings at 6 dpf plotted per condition over the 20-50 Hz region for *sv2a^+/+^* and *sv2a^–/–^* larvae after incubation with levetiracetam and valproic acid at their MTC or DMSO control at 5 dpf for 22 h. Number of larvae per condition: n = 9–35. Statistical analysis was performed using ordinary one-way ANOVA. **p < 0.01, ***p < 0.001, ****p < 0.0001.

To investigate whether *sv2a* knockout resulted in abnormal brain activity, we recorded LFP on larval optic tecta of 6 dpf *sv2a^–/–^*, *sv2a^+/–^*, and *sv2a^+/+^* larvae. Epileptiform events consisted of polyspiking bursts with amplitudes equal to or exceeding threefold the baseline (Figure 3A). To quantify these events, we performed a PSD analysis by computing average power in consecutive 10 Hz frequency bands ranging from 1 to 150 Hz and normalized against *sv2a^+/+^* larvae (Figure 3B). *sv2a^–/–^* larvae had a significantly higher PSD within the 20 and 50 Hz frequency range (Figure 3B), confirming the frequency band characterizing polyspiking discharges (Schubert et al., 2014). PSDs, plotted as mean PSD per condition over the 20–50 Hz region (Figure 3C), confirmed that there was a significant increase (*p* < 0.05) in the PSD values for *sv2a^–/–^* larvae in comparison to *sv2a*^+/–^ and *sv2a^+/+^* siblings. The morphological, behavioral, and epileptic phenotype of *sv2a^–/–^* CRISPR was fully recapitulated and confirmed in the *sv2a* morphants generated by an antisense morpholino knockdown (Supplementary Figure 2).

To determine whether 6 dpf *sv2a^–/–^* larvae showed differences in the brain organization, a histological analysis was performed by hematoxylin and eosin staining. The optic nerve was chosen as a reference point to obtain comparable sections per genotype. We found that there were significantly less hematoxylin-positive stained nuclei detected in the pallium (midbrain) of *sv2a^–/–^* larvae than in those of their *sv2a*^+/–^ and *sv2a^+/+^* siblings (Figure 3D). However, additional examination of the brain showed no gross morphological abnormalities in *sv2a^–/–^* brains. As shown in Figure 3E, on the example of the telencephalic section of *sv2a*-deficient zebrafish larva, there were no gross structural changes, developmental aberrations, or differences in the formation of characteristic brain areas including the telencephalon, pallium, preoptic region, and thalamic structures.

### Effects of Anti-epileptic Drugs on Seizures of *sv2a* Knockout Zebrafish

We then tested anti-seizure effects of sodium valproate and levetiracetam, commonly used AEDs in epilepsy treatment, in the LFP assay on 6 dpf *sv2a^–/–^* mutant larvae. Noteworthy, SV2A is the distinct binding site for levetiracetam (Lynch et al., 2004). Our results demonstrated that sodium valproate substantially reduced epileptiform brain activity (Figure 3F) to 35% (*p* < 0.001). Surprisingly, treatment with levetiracetam also showed a significant effect on the mean PSD values by reducing the epileptiform events to 40% (*p* < 0.01) in *sv2a^–/–^* larvae. This strongly suggests that other targets besides SV2A must be involved in levetiracetam’s mechanism of action for seizure suppression.

### Characterization of *sv2a^–/–^* Larval Transcriptome Reveals That *sv2a* Knockout Alters Gene Expression in Developing Larval Brains

To obtain mechanistic insights into the consequence of the *sv2a* absence in developing larval brains, RNA sequencing was performed on the heads of *sv2a^–/–^*, *sv2a*^+/–^, and *sv2a^+/+^* 6 dpf larvae. Approximately 37.4 million single-end reads were produced per sample. After quality assessment and filtering, over 37.1 million remained, of which 92.6% aligned to the zebrafish reference genome GRCz11. Overall, 28,484 genes were expressed in at least one of the three genotypes. While the majority of expressed genes was protein-coding (85.9%), long non-coding RNAs (6.5%), non-coding RNAs (3.7%), and pseudogenes (0.8%) were detected as well. The remaining percentages of expressed genes categorized to a variety of other RNAs species (Supplementary Table 2).

**FIGURE 4:**
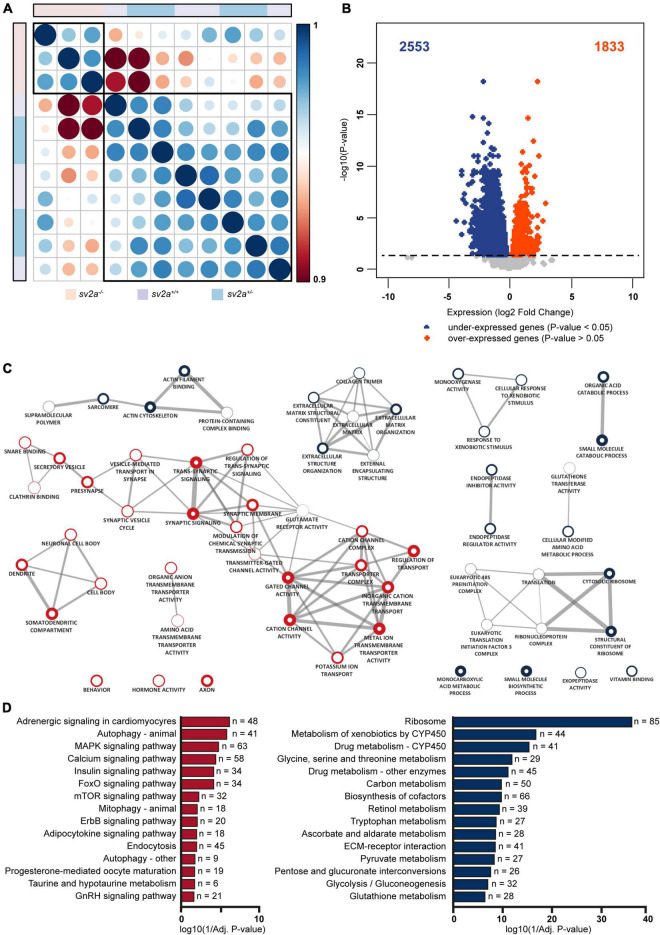
Transcriptome analysis and gene ontology (GO) enrichment map of *sv2a^–/–^* larvae. **(A)** Spearman’s rank correlation matrix of the RNA-Seq data showing separate clustering of *sv2a^–/–^* samples from *sv2a^+/+^* and *sv2a*^+/–^ samples. Scale bar indicates the strength of the correlation, with 1 indicating a strong positive correlation (dark blue) and 0.9 indicating weak correlation (dark red) between samples. Areas of the circles show absolute values of corresponding correlation coefficients. Samples of different genotypes are indicated in distinct colors codes. **(B)** Volcano plot showing the differentially expressed genes (DEG) (padj < 0.05) between *sv2a^–/–^* and *sv2a^+/+^* larvae. Of the 4386 DEGs, 2553 were found to be down- (blue) and 1833 to be up-regulated (red) in *sv2a^–/–^* larvae compared to *sv2a^+/+^*. **(C)** GO enrichment map from up- and down-regulated genes between *sv2a^+/+^* and *sv2a^–/–^* zebrafish larvae. Each node represents a different GO term, the red and blue outside of nodes indicate enrichment in up- or down-regulated genes, respectively. The larger the node the greater the number of genes in the enriched GO term. Connecting lines indicate common genes shared between nodes, the thicker the line the more genes in common. **(D)** Enriched pathways from differentially expressed genes between *sv2a^–/–^* and *sv2a^+/+^* zebrafish larvae. Pathways enriched amongst up-regulated genes are indicated in red, pathways enriched amongst down-regulated genes are indicated in blue. The x-axis represents the log10 (1/*p*-value), n indicates the number of genes appearing in each category.

Both principal component analysis (PCA) and t-distributed stochastic neighborhood embedding (t-SNE) of the gene expression profiles revealed that two divergent groups could be distinguished (Supplementary Figures 3A,B), which was confirmed by a Spearman’s correlation matrix of the gene expression showing that the *sv2a^–/–^* samples clustered separately from their *sv2a*^+/–^ and *sv2a^+/+^* siblings (Figure 4A). Further reflecting this grouping, analysis of the gene expression profiles of *sv2a^+/+^* and *sv2a*^+/–^ samples revealed only one differentially expressed gene (DEG), namely *sv2a*. In contrast, between *sv2a^+/+^* and *sv2a^–/–^*, and *sv2a*^+/–^ and *sv2a^–/–^*, there were 4386 and 3535 DEGs, respectively (Figure 4B and Supplementary Figure 3C). Of the 4386 DEGs between *sv2a^+/+^* and *sv2a^–/–^*, 1833 (135 with absolute fold change > 2) were up- and 2553 (990 with absolute fold change > 2) were down-regulated in *sv2a^–/–^* larvae. Top ten up- and down-regulated protein-coding genes sorted by Wald statistic are listed in Table 1 (complete list in Supplementary Table 2). To validate the RNA-seq data, a set of five up- and five down-regulated genes, including gonadotropin-releasing hormone 3 *(gnrh3)*, FOS like 1, AP-1 transcription factor subunit a *(fosl1a)*, v-fos FBJ murine osteosarcoma viral oncogene homolog Ab *(fosab)*, activating transcription factor 3 *(atf3)*, transmembrane protein 176l.3a *(tmem176l.3a)*, ATP-binding cassette, sub-family G (WHITE), member 2b (*abcg2b)*, four and a half LIM domains 5 *(fhl5)*, fatty acid binding protein 1b, tandem duplicate 1 *(fabp1b.1)*, nuclear receptor subfamily 0, group B, member 2a *(nr0b2a)* and apolipoprotein Da, duplicate 1 (*apoda.1)*, was selected for RT-qPCR analysis in a cohort of three *sv2a^–/–^* and three *sv2a^+/+^* samples. Significant differences in the expression levels were observed for the up-regulated genes *fosl1a* and *atf3*, and down-regulated genes *abcg2b, fhl5, fabp1b.1, nr0b2a*, and *apoda.1*. A non-significant up-regulated trend was detected for *gnrh3, fosab*, and *tmem176l.3a*. In conclusion, the experimental expression pattern of these up- and downregulated genes was in line with the results obtained by RNA sequencing (Supplementary Figure 3D).

**TABLE 1:** Top 10 up- and down-regulated genes sorted by Wald statistic.

Ensemble ID	Gene name	Fold change	Chromosome	Wald statistic	Adjusted *p*-value
**Top 10 up-regulated protein coding genes**					
ENSDARG00000018744	prl2	4.81	4	9.83	7.39E-19
ENSDARG00000045016	npffl	2.73	11	8.90	2.37E-15
ENSDARG00000056214	gnrh3	3.79	17	8.22	4.98E-13
ENSDARG00000014927	uts1	2.13	20	7,89	4.60E-12
ENSDARG00000055752	npas4a	5.18	14	7.72	1.27E-11
ENSDARG00000024831	crhbp	1.93	21	7.44	9.13E-11
ENSDARG00000069089	agrp	3.03	7	7.40	1.16E-10
ENSDARG00000023217	crema	2.44	2	7.31	2.18E-10
ENSDARG00000027657	crhb	2.00	24	7.14	4.82E-10
ENSDARG00000031336	hsd20b2	2,67	5	7.12	5.62E-10
**Top 10 down-regulated protein coding genes**					
ENSDARG00000059945	sv2a	0.16	16	−16.71	2.54E-58
ENSDARG00000070038	rbp2a	0.23	15	−9.81	7.39E-19
ENSDARG00000095751	leg1.2	0.12	20	−8.93	2.23E-15
ENSDARG00000090268	krtt1c19e	0.22	19	−8.86	2.91E-15
ENSDARG00000060345	apoda.1	0.30	2	−8.72	8.53E-15
ENSDARG00000070029	ehhadh	0.28	9	−8.46	7.35E-14
ENSDARG00000044685	nr0b2a	0.22	16	−8.20	5.36E-13
ENSDARG00000013755	actn3a	0.35	21	−8.04	1.83E-12
ENSDARG00000017388	gstt1b	0.41	21	−7.97	2.93E-12
ENSDARG00000062688	gpnmb	0.40	19	−7.95	3.23E-12

*Ensemble ID, gene name, absolute fold change, chromosome number, Wald statistic, and adjusted p-value are printed per each gene.*

To understand how zebrafish larvae lacking *sv2a* compare with other previously established zebrafish epilepsy models, the *sv2a^–/–^* transcriptome was compared to the transcriptomes of five zebrafish epilepsy models: *gabra1* (Samarut et al., 2018) recapitulating idiopathic generalized epilepsy, *depdc5* (Swaminathan et al., 2018) and *tsc2* (Scheldeman et al., 2017) modeling mTORopathies, *scn1lab* (unpublished data) recapitulating Dravet syndrome, and kainic acid-injected (unpublished data), a chemically-induced larval epilepsy model. Compared to the other genetic epilepsy models, *sv2a^–/–^* zebrafish displayed a considerably greater number of DEGs. In addition, none of the five models demonstrated a substantial overlap of DEGs with the *sv2a^–/–^* model (Supplementary Figure 3E). These observations indicate that the mechanisms involved in the *sv2a* loss-of-function zebrafish epilepsy model at least partly differ from those observed in the other zebrafish epilepsy models and therefore highlight the uniqueness of this model.

GO enrichment analysis demonstrated a substantial amount of GO terms enriched in both the up- and down-regulated genes between *sv2a^–/–^* and wildtype zebrafish larvae (Supplementary Table 3). Enrichment Map analysis of the top 10 GO terms (Biological Process, Molecular Function or Cellular Component) amongst up-regulated genes revealed one major cluster containing terms related to synapses and ion channels and one smaller cluster containing terms related to neurons (Figure 4C). For the down-regulated genes there were four smaller clusters identified, including one that contained terms related to extracellular matrix (ECM) and another with terms related to translation (Figure 4C). Organic anion transport was the only common enriched GO term shared by the up- and down-regulated gene lists. The larger number of down-regulated genes compared to up-regulated genes was reflected in the total number of significantly enriched GO terms (*p*-value < 0.05), which was larger amongst down-regulated genes (202 terms amongst down-regulated genes versus 165 amongst up-regulated genes).

The pathway analysis identified 20 enriched pathways (*p*-value < 0.05) from the up-regulated genes (Supplementary Table 3). Three interesting pathways amongst the top 10 of this list were MAPK signaling, calcium signaling and mTOR signaling (Figure 4D). Amongst the down-regulated genes there were 40 enriched pathways (*p*-value < 0.05) (Figure 4D and Supplementary Table 3) and noteworthy pathways from this list were glycolysis/gluconeogenesis and ECM-receptor interaction.

### Connectivity Mapping Correctly Predicts Therapeutic Compounds Capable of Reverting the *sv2a^–/–^* Epileptic Phenotype

**FIGURE 5:**
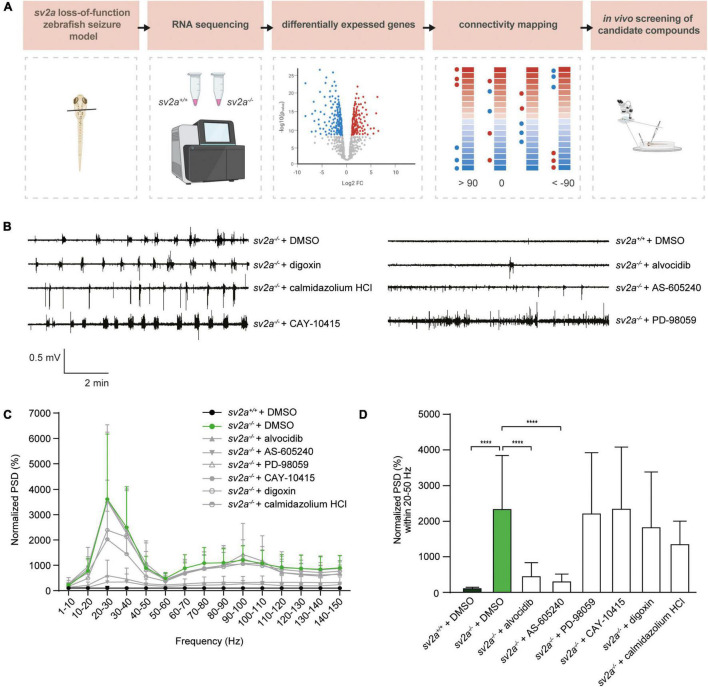
Connectivity mapping in *sv2a^–/–^* zebrafish larvae. **(A)** Overview of the connectivity mapping workflow. Heads of *sv2a^–/–^* and *sv2a^+/+^* zebrafish larvae were sampled and RNA was extracted. After RNA sequencing and differential expression analysis, top 50 up- and down-regulated genes in *sv2a^–/–^* larvae (compared to *sv2a^+/+^*) were selected based on Wald statistic and compared to gene expression profiles of compounds in the CMap database in order to identify compounds reversing the differential expression signature (scores < −90). Three of these compounds (alvocidib, AS-605240, PD-98059) and three negative controls (scores > 90; digoxin, calmidazolium HCl, or equaling zero; CAY-10415) were tested in the *sv2a* loss-of-function in LFP recordings. **(B)** Representative LFP recordings of *sv2a^–/–^* and *sv2a^+/+^* larvae after incubation with selected CMap compounds at their MTCs or DMSO control at 5 dpf for 22 h. **(C)** PSD analysis of *sv2a^–/–^* and *sv2a^+/+^* larvae after incubation of selected CMap compounds at their MTCs or DMSO control at 5 dpf for 22 h. Results were normalized to *sv2a*^+/+^ larvae as 100%. Number of larvae per condition: *n* = 9–27. **(D)** Power spectral density (PSD) values (mean ± SD) from LFP recordings at 6 dpf plotted per condition over the 20-50 Hz region for *sv2a^–/–^* and *sv2a^+/+^* larvae after incubation of selected CMap compounds at their MTCs or DMSO control at 5 dpf for 22 h. Number of larvae per condition: *n* = 9–27. Data are presented as mean ± SD. Statistical analysis was performed using ordinary one-way ANOVA. ***p < 0.001, ****p < 0.0001.

In order to explore the potential of connectivity mapping for the discovery of novel anti-epileptic drugs, we employed the CMap database to identify substances with gene signatures opposed the one observed in *sv2a^–/–^* zebrafish larvae (Figure 5A). To this end, a list of up- and down-regulated genes selected by Wald statistic was used to query the library (see materials and methods for more details). Twenty-one compounds were found to have highly opposing scores (<−90), of which three were selected for further testing (Table 2). The overall greatest negative connectivity score was found for PD-98059, a potent mitogen-activated protein kinase (MEK) inhibitor (Dudley et al., 1995), being very valuable in the elucidation of the MAPK signaling pathway in different immune responses (Guha et al., 2001; Reiling et al., 2001). Additionally, we selected alvocidib (formerly flavopiridol), a cyclin-dependent kinase (CDK) inhibitor developed for the treatment of acute myeloid leukemia (Zeidner and Karp, 2015) and AS-605240, a potent and selective phosphoinositide 3-kinase (PI3K) gamma inhibitor that never progressed into clinical development but is still commonly used for *in vitro* research purposes. We also included several negative controls: compounds that theoretically should not interfere with or worsen the *sv2a^–/–^* disease phenotype (CAY-10415, and calmidazolium HCl and digoxin, respectively) (Table 2).

**TABLE 2:** Overview of compounds identified with CMap and selected for further testing.

Compound name	Description	Connectivity score
PD-98059	MEK inhibitor	−99.08
AS-605240	PI3K inhibitor	−93.64
Alvocidib	CDK inhibitor	−91.48
CAY-10415	Insulin sensitizer	0
Calmidazolium HCl	Calcium channel blocker	96.39
Digoxin	ATPase inhibitor	96.44

*Description indicates the suggested mechanism of action. Negative scores indicate highly opposing gene signatures, positive scores indicate highly similar gene signatures. A compound score of 0 indicates no correlation between compound and disease signature at all.*

5 dpf *sv2a*^–/–^ zebrafish larvae were treated with the selected compounds at their MTCs for 22 h (Figures 5B–D). At 6 dpf, LFP recordings were performed to determine if the identified compounds were able to decrease epileptiform brain activity. Interestingly, two out of three compounds with opposing gene signatures (alvocidib and AS-605240) were able to significantly decrease epileptiform brain discharges in *sv2a*^–/–^ zebrafish larvae, while all three negative controls did not significantly affect the PSD values. Since incubation in separate wells for 22 h resulted in a loss of the observed hyperactivity in *sv2a*^–/–^ larvae, we were not able to investigate behavioral effects of these compounds using the automated high-throughput tracking system.

## Discussion

In the current study, we report the generation and characterization of a novel *sv2a* knockout zebrafish model using CRISPR/Cas9 and the application of connectivity mapping in finding novel drug candidates against epilepsy. To summarize, the absence of *sv2a* in knockout larvae resulted in premature death, locomotor hyperactivity, and severe spontaneous electrographic seizures. Meanwhile, their *sv2a*^+/–^ and *sv2a^+/+^* siblings developed into fertile adults and did not show any abnormalities in their behavior or brain activity. Therefore, the knockout larvae recapitulate phenotypic features typically observed in the *SV2A* null mice models (Crowder et al., 1999; Janz et al., 1999; Kaminski et al., 2009; Menten-Dedoyart et al., 2016) as these mice failed to grow, developed severe seizures, and resulted in early death. On the contrary, partial SV2A protein deficiency in heterozygous mice did not have any impact on the lifespan or basic sensimotor functions (Kaminski et al., 2009; Lamberty et al., 2009). Even though Sv2a is essential for survival and proper functioning of the nervous system, no gross brain malformations or differences in the formation of characteristic brain areas could be observed in *sv2a^–/–^* larvae. These findings again are in line with those of *SV2A* knockout mice, where the overall brain architecture, including the cerebral cortex, hippocampus, and thalamus, was similar to wildtype animals (Crowder et al., 1999; Janz et al., 1999), indicating that deletion of *sv2a* does not lead to developmental changes in brain structure. Loss of *sv2a* expression in *sv2a^–/–^* larvae resulted, however, in a substantial decrease of cell nuclei in the pallium region, but the underlying cause of this neuronal loss remains to be elucidated. Therefore, the epileptic phenotype is likely an effect of the modulation of neurotransmitter levels at the synapses.

It is well known that the GABA excitatory to inhibitory switch (E-I switch) is a hallmark of the developing nervous system (Ben-Ari et al., 1989; Ben-Ari et al., 2007; Zhang et al., 2010; Momose-Sato et al., 2012; Lysenko et al., 2018; Kang et al., 2019). In a previous study a rapid increase of *SV2A* expression in the CA1 region of mouse hippocampus was observed at P7, which is the time point when the GABA E-I switch is completed (Valeeva et al., 2013). This might explain why the absence of *SV2A* is becoming dominant on GABAergic inhibition only after P7, which is precisely the age when *SV2A* knockout mice start to have seizures (Crowder et al., 1999). In zebrafish, the period between 2 and 4 dpf has been proven to be critical for the E–I switch of GABAergic action (Zhang et al., 2010). Interestingly, we found that the *sv2a* expression gradually increased and stabilized around 4 dpf. In fact, epileptiform brain activity could already be detected in 5 dpf *sv2a^–/–^* larvae (Supplementary Figure 4), which is in line with *SV2A* knockout mice studies. However, since in zebrafish the major organs including the brain are more developed after 5 dpf (Garcia et al., 2016), therefore we employed more mature 6 dpf larvae to study Sv2a function.

Unexpectedly, epileptiform activity in *sv2a^–/–^* larvae could be attenuated by treatment with levetiracetam, even though there was nearly a complete absence of its presumed target, as confirmed by low levels of *sv2a* via RT-qPCR. Previous studies have demonstrated decreased anticonvulsant efficacy of levetiracetam observed in *SV2A* heterozygous 6-Hz mice model, which was consistent with less binding sites available (Kaminski et al., 2009). Moreover, it has been previously shown that *SV2A* expression was reduced during epileptogenesis and in the chronic epileptic phase in rats, and throughout the hippocampus of TLE patients (van Vliet et al., 2009). It has not been investigated, however, whether this affected the effectiveness of levetiracetam. Interestingly and in line with our results, *SV2A*-caused photosensitive reflex epilepsy chicken model was sensitive to levetiracetam since the drug (partially) rescued the epileptic phenotype, although the levels of *SV2A* were shown to be very low in homozygous animals (Douaud et al., 2011). Hence the unanticipated finding that levetiracetam exerted antiseizure activity in *sv2a^–/–^* larvae, inconsistent with the absence of its presumed target, strongly suggests that levetiracetam may act (at least partly) on other target(s) than Sv2a in order to exert its antiepileptic activity.

Although levetiracetam is a broad-spectrum AED with neuroprotective properties and is widely used to treat focal onset and generalized seizures, the exact detail of the mechanism of action of levetiracetam is not clear. Unquestionably, levetiracetam binds to SV2A to modulate neurotransmitter release including GABA and glutamate (Lynch et al., 2004), which supports the evidence that this mechanism is important for the anticonvulsant properties of the drug (Kaminski et al., 2009). Nevertheless, levetiracetam (unlike its more potent analog brivaracetam) has only moderate affinity to SV2A and possesses several other putative pharmacologic mechanisms of action (Klitgaard et al., 2016). It has been demonstrated that levetiracetam inhibited presynaptic calcium channels through an intracellular pathway (Vogl et al., 2012) and also acted as an AMPA receptor antagonist (Carunchio et al., 2007; Vogl et al., 2012; Steinhoff and Staack, 2019). These pathways could contribute to the anticonvulsant effects of levetiracetam by reducing neuronal excitability. Moreover, it was shown that the major metabolite of levetiracetam inhibited histone deacetylase and induced histone hyperacetylation in human cells (Eyal et al., 2004). Modulation of chromatin structure through histone modifications is an important regulator of gene transcription in the brain and altered histone acetylation seems to contribute to changes in gene expression associated with epilepsy and epileptogenic processes (Citraro et al., 2017). Additional investigations have also revealed that levetiracetam could induce embryonic neurogenesis *in vitro* by increased cell proliferation and differentiation of rat embryonic neural stem cells, mainly through an NMDA receptor-mediated mechanism (Alavi et al., 2021). Interestingly, activated microglia, that maintain immune homeostasis in the brain, contribute to the changes occurring during epileptic processes (Victor and Tsirka, 2020). Levetiracetam inhibited microglial activation by suppressing excess microglial phagocytosis during epileptogenesis (Itoh et al., 2019), which might prevent the occurrence of spontaneous recurrent seizures. More recently, it has been demonstrated that levetiracetam significantly suppressed the inflammatory reactions in microglial cells that did not express *SV2A*, and FosL1, activator protein 1(AP-1) transcription factor subunit, was identified as the most likely target of levetiracetam responsible for this anti-inflammatory mechanism (Niidome et al., 2021). All the above-described effects may contribute to the unique pharmacological profile of levetiracetam, suggesting that binding to SV2A might not represent its primary mechanism of action. Alternatively, as our CRISPR knockout did not result in a complete loss of *sv2a* mRNA and because of the absence of a seizure phenotype in *sv2a*^+/–^ zebrafish larvae and studies in *SV2A* heterozygous mice showing that full restoration is not required for phenotype recovery, we cannot exclude that the observed effects of levetiracetam are resulting from its action on residual Sv2a protein levels. Unfortunately, due to the lack of zebrafish-specific and cross-reacting antibodies it was not possible to further investigate this hypothesis.

To gain more insight into the molecular basis underlying the observed epileptic phenotype in *sv2a* knockout larvae, we further investigated differences between the distinct genotypes at the transcriptome level, which is to the best of our knowledge the first report of a transcriptome study in a *sv2a* deleterious animal model. Large perturbations of the *sv2a^–/–^* larval transcriptome were observed when compared to their wildtype siblings. Many of the DEGs were involved in synapses, ion channels, neurons, and ECM, demonstrating that our *sv2a* loss-of-function zebrafish model mimics aspects of human epilepsy (Okamoto et al., 2010; Winden et al., 2011; Pitkanen et al., 2015; Engel and Pitkanen, 2020). For example, although molecular mechanisms of epilepsy are not well understood and differ amongst distinct patient populations, dysregulation of proteins determining excitability, including ion channels and GABA receptors, are commonly observed (Snowball and Schorge, 2015; Akyuz et al., 2021). Additionally, it is known that ECM are linked to synaptic reorganization and are often seen in epileptogenic zones in patients (Leite and Peixoto-Santos, 2021). Other interesting pathways enriched amongst differentially expressed genes in *sv2a^–/–^* larvae were MAPK-, mTOR-, and calcium signaling and their involvement in epileptogenesis has been previously confirmed (Steinlein, 2014; Pernice et al., 2016; Scheldeman et al., 2017). Despite the observation that our *sv2a* knockout model shares mechanisms common for epilepsy, a comparison with the transcriptome profiles of five other established larval zebrafish epilepsy models did not show any large overlap of DEGs with the other models. This finding implies that the mechanisms involved in the *sv2a* loss-of-function model at least partly differ from those observed in the other zebrafish epilepsy models, highlighting not only the novelty and uniqueness of our model, but also its potential use for drug screening to find AEDs with novel mechanisms of action.

Accordingly, we applied the concept of connectivity mapping as a novel drug discovery strategy in our *sv2a* zebrafish model. We demonstrated that two predicted candidates, AS-605240 and alvocidib, were able to significantly decrease epileptiform brain discharges in the *sv2a^–/–^* larvae. AS-605240 is an effective PI3Kγ inhibitor (Ksionda et al., 2018). The PI3K/Akt/mTOR pathway is essential for intracellular signaling, regulating neuronal survival, growth, and plasticity (Switon et al., 2017) and, importantly, is known to be overactivated after epileptogenic brain injury in animal models as well as in patients with acquired epilepsies (Vezzani, 2012). Alvocidib on the other hand, is known to inhibit CDK (Zeidner and Karp, 2015). Interestingly, it was shown that CDK5 plays important neuronal function and is crucial for homeostatic synaptic plasticity. Moreover, association between chronic loss of CDK5 and presence of seizures has been reported in other animal epilepsy models (Dixit et al., 2017). Although PI3K and CDK are plausible anti-epileptic targets of the identified candidates, further elucidation of the mechanism of action of AS-605240 and alvocidib is recommended. Overall, the results achieved in this explorative study clearly show that connectivity mapping using zebrafish transcriptome data as an input is reliable and effective, as we experimentally confirmed two out of three positively as well as three out of three negatively predicted therapeutic candidates. In addition, we demonstrated that preceding a traditional phenotypical drug screen with a network-based computational analysis tremendously improves observed hit rates and therefore reduces time and cost spent on a typical drug screening. However, one drawback of the connectivity mapping strategy is its tendency to be very variable, since no consensus input dataset neither threshold connectivity value exists. For future research, a more defined design and fixed methodology might increase reproducibility and less bias in the promising field of network-based drug screening. In this context, it is also important to note that the concept of connectivity mapping, which relies on ameliorating an observed disease phenotype by reversing a dysregulated gene expression pattern, differs from the traditional drug discovery concept that aims at finding (ant)agonists that functionally act on protein-based targets. As such, one cannot exclude the possibility that drugs that are not identified in the CMap analysis, and thus do not reverse the dysregulated gene expression profile, have disease-ameliorating effects.

Collectively, we demonstrated that the absence of *sv2a* resulted in an increased seizure susceptibility and provided important insight into the functional relevance of *sv2a* in the brain in general. Additionally, since *sv2a^–/–^* larvae, lacking the presumed target of levetiracetam, were responsive to this AED, the model is a great asset to the field for investigating its putative targets. In addition, given the fact that the *sv2a^–/–^* differentially expressed gene dataset did not show large overlap with datasets of other larval epilepsy models, our *sv2a* loss-of-function zebrafish model could be of great value in the search for anti-epileptics with novel mechanisms of action. Specifically, we supplied evidence that the concept of connectivity mapping represents an attractive and powerful strategy in the discovery of novel AEDs.

## Data Availability Statement

The datasets presented in this study can be found in online repositories. The names of the repository/repositories and accession number(s) can be found in the article/Supplementary Material.

## Ethics Statement

The animal study was reviewed and approved by Ethics Committee of the KU Leuven (P023/2017 and P027/2019) and by the Belgian Federal Department of Public Health, Food Safety, and Environment (LA1210199).

## Author Contributions

YZ, LH, MP, MG, and AS performed the zebrafish experiments and analyzed the data. LH, JM, and PG analyzed the transcriptome data. YZ, LH, and AS designed the experiments. PW and RK conceived the study. PW and AS supervised the study. YZ, LH, PW, and AS wrote the manuscript. All authors contributed to the article and approved the submitted version.

## Conflict of Interest

RK, PG, and MG were employed at UCB Pharma at the time this study was performed. The remaining authors declare that the research was conducted in the absence of any commercial or financial relationships that could be construed as a potential conflict of interest.

## Publisher’s Note

All claims expressed in this article are solely those of the authors and do not necessarily represent those of their affiliated organizations, or those of the publisher, the editors and the reviewers. Any product that may be evaluated in this article, or claim that may be made by its manufacturer, is not guaranteed or endorsed by the publisher.

## Footnotes

1
www.nucleomics.be

2
http://www.ebi.ac.uk/arrayexpress

3
http://www.bioconductor.org

4
http://www.cytoscape.org/

## Supplementary Material

Supplementary Table 1 Overview qPCR and HRM primers.

Supplementary Table 2 DEG list.

Supplementary Table 3 Enriched GO terms and KEGG pathways.
