# Supplementary material for: Connectivity Mapping Using a Novel sv2a Loss-of-Function Zebrafish Epilepsy Model as a Powerful Strategy for Anti-epileptic Drug Discovery
Source: Front Mol Neurosci. 2022 May 24;15:881933. doi: 10.3389/fnmol.2022.881933 (PMC9172968; doi:10.3389/fnmol.2022.881933)
Supplement: Supplementary file 4 [file Image_1.PDF]

A

```
zebrafish   MDEGYRDRSAFIKGAKDIAKEVKRHAGKKVGRHVDKMTDEYTKRSYTRFEEDDDDDDYPV   60
human       MEEGFRDRAAFIRGAKDIAKEVKKHAAKKVVKGLDRVQDEYSRRSYSRFEEEDDDDDFPA   60
            *:**:***:***:*********:**.*** : :*:: ***::***:****:*****:*.

zebrafish   QAQDGSYYRSNSRANDDEGAHSDSTEGHDEDDEIYEGEYQGIPRNDSVEKADRQVGAVGQ   120
human       PS-DGYYRGEGTQDEEEGGASSDATEGHDEDDEIYEGEYQGIPRAESGGKGERMADGAPL   119
             : ** *  ..:: ::: ** **:******************** :*  *.:* ....

zebrafish   S---------QFRDMTQYEGERRKDQEELAQQYETILQECGHGRFQWTLYFVLGLALMAD   171
human       AGVRGGLSDGEGPPGGRGEAQRRKEREELAQQYEAILRECGHGRFQWTLYFVLGLALMAD   179
            :          :     : *.:***::********:**:***********************

zebrafish   GVEIFVVGFVLPSAEKDMCLSEPNKGMLGLIVYLGMMVGAFVWGGLADRIGRRQTLLISL   231
human       GVEVFVVGFVLPSAEKDMCLSDSNKGMLGLIVYLGMMVGAFLWGGLADRLGRRQCLLISL   239
            ***:*****************: *******************:*******:**** *****

zebrafish   SINSVFAFFSSFVQGYSSFLFCRLLSGVGIGGSIPIVFSYYSEFLAQEKRGEHLSWLCMF   291
human       SVNSVFAFFSSFVQGYGTFLFCRLLSGVGIGGSIPIVFSYFSEFLAQEKRGEHLSWLCMF   299
            *:**************.:**********************:*******************

zebrafish   WMIGGIYAAAMAWAIIPHYGWSFQMGSAYQFHSWRVFVLVCAFPSVAAIAALTTMPESPR   351
human       WMIGGVYAAAMAWAIIPHYGWSFQMGSAYQFHSWRVFVLVCAFPSVFAIGALTTQPESPR   359
            *****:*************************************** **.**** *****

zebrafish   FYLENGKHDEAWMILKQVHDTNMRAKGYPERVFSVTTIKTVKQMDELVDMGGEATAWHQR   411
human       FFLENGKHDEAWMVLKQVHDTNMRAKGHPERVFSVTHIKTIHQEDELIEIQSDTGTWYQR   419
            *:***********:*************:******** ***::* ***::: .:: :*:**

zebrafish   WRIKLTNLFHQVWGNFLTVFNPEYRRITYMMMAVWFSMSFSYYGLTVWFPDMIKYLQKQE   471
human       WGVRALSLGGQVWGNFLSCFGPEYRRITLMMMGVWFTMSFSYYGLTVWFPDMIRHLQAVD   479
            * ::  .*  *******: *.******* ***.***:*****************::**  :

zebrafish   YSSRTKVFIKEKVEHVTFNFTLENQIHRNGEYFNNKFLNLKMKSMVFEDSLFEECYFEDI   531
human       YASRTKVFPGERVEHVTFNFTLENQIHRGGQYFNDKFIGLRLKSVSFEDSLFEECYFEDV   539
            *:******  *:****************.*:***:**:.*::**: *************:

zebrafish   TSSNTFFRNCTFISTLFYNTDLFKYRLINSKLINSTFLHNKEGCMLDFSDE-NNAYMIYF   590
human       TSSNTFFRNCTFINTVFYNTDLFEYKFVNSRLINSTFLHNKEGCPLDVTGTGEGAYMVYF   599
            *************.*:*******:*::**:************* **.:.  :.***:**

zebrafish   VSFLGTLAVLPGNIVSALLMDKIGRLRMLAGSSVISCVSCFFLSFGNSESAMIALLCLFG   650
human       VSFLGTLAVLPGNIVSALLMDKIGRLRMLAGSSVMSCVSCFFLSFGNSESAMIALLCLFG   659
            **********************************:*************************

zebrafish   GISIASWNALDVLTVELYPSDKRTTAFGFLNALCKLAAVLGISIFQSFVGITKAVPILFA   710
human       GVSIASWNALDVLTVELYPSDKRTTAFGFLNALCKLAAVLGISIFTSFVGITKAAPILFA   719
            *:*************************************** ********.*****

zebrafish   SGALAAGSFLALKLPETRGQVLQ 733
human       SAALALGSSLALKLPETRGQVLQ 742
            *.*** ** **************
```

B

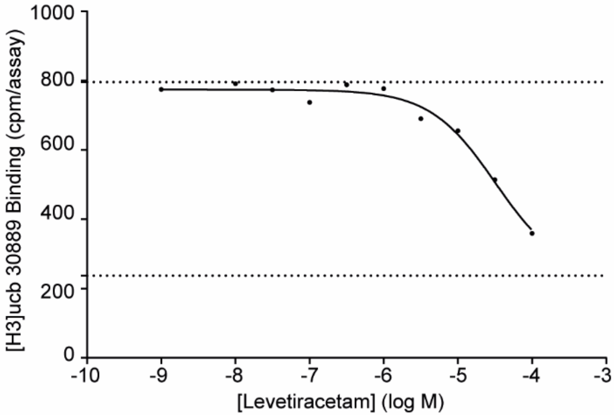

Figure S1. (A) Amino acid sequence alignment of the zebrafish Sv2a and human SV2A protein using Clustal Omega (Madeira et al, 2019). Important ligand binding residues include C297, W300, Y462, W666, N667, and D670 (Lee et al, 2015), and are indicated in red. (B) Affinity of levetiracetam for Sv2a in HEK293 cells expressing the recombinant protein. Levetiracetam was incubated at increasing concentrations of [3H]ucb 30889 for 60 min at 37 °C as described previously by Gillard et al, 2006; Gillard et al, 2011 and Wood et al, 2020. SD was in the range of 5% to 10% of the mean but was omitted in the graph for clarity. The pIC50 was determined to be 4.5, in line with values reported for recombinant and native human and rat SV2A (Gillard et al, 2006; Gillard et al, 2011; Wood et al, 2020).
