# Supplementary material for: Connectivity Mapping Using a Novel sv2a Loss-of-Function Zebrafish Epilepsy Model as a Powerful Strategy for Anti-epileptic Drug Discovery
Source: Front Mol Neurosci. 2022 May 24;15:881933. doi: 10.3389/fnmol.2022.881933 (PMC9172968; doi:10.3389/fnmol.2022.881933)
Supplement: Supplementary file 5 [file Image_2.pdf]

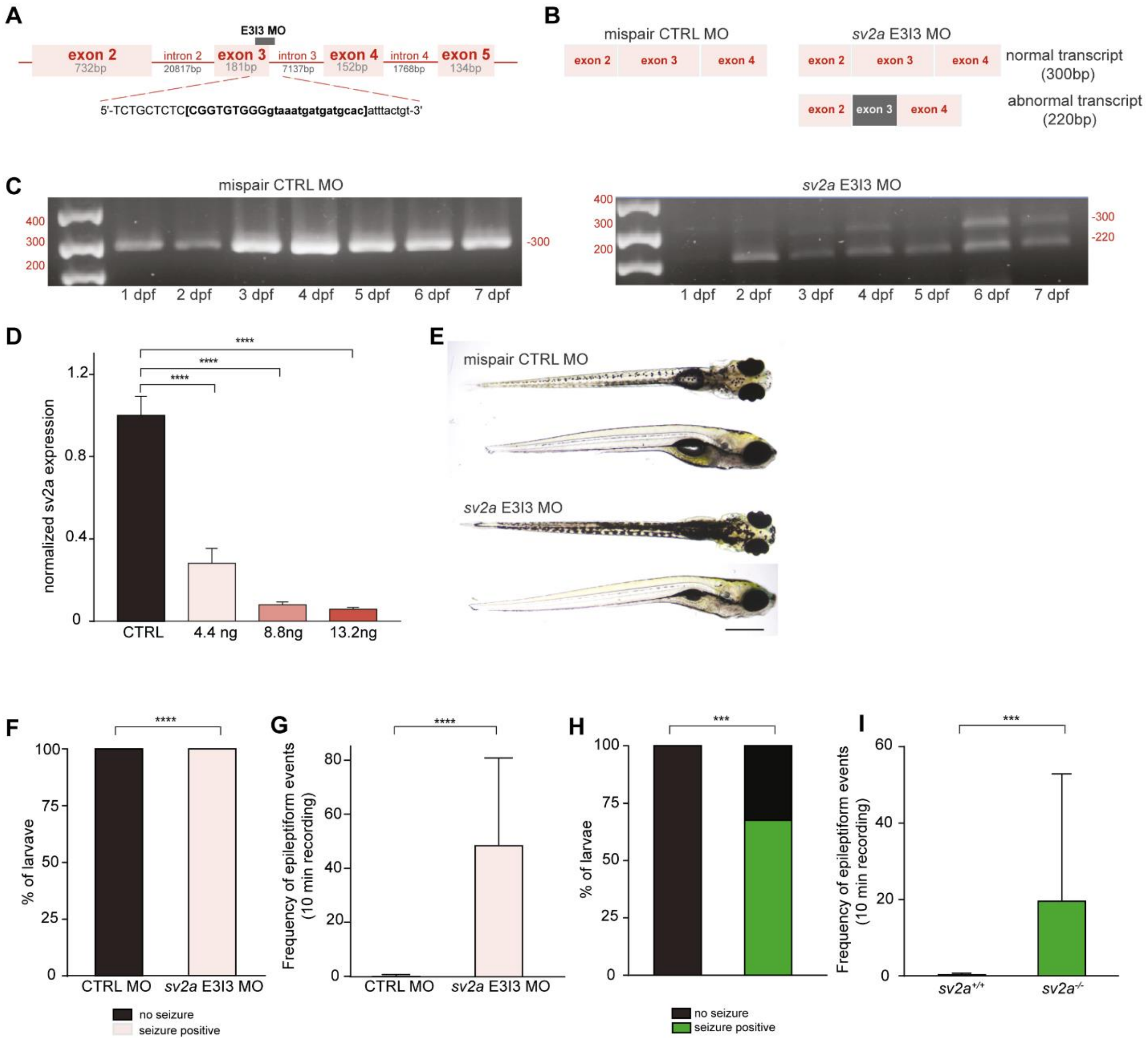

Figure S2. *sv2a* morphants generated by an antisense morpholino (MO) knockdown recapitulate morphological and epileptic phenotype of *sv2a*$^{-/-}$ larvae. (A) Schematic view of the first coding exons (pink boxes) and introns (black lines) of zebrafish *sv2a* with splicing-inhibiting exon 3-intron 3 (E3I3)-*sv2a* MO (grey box) targeting splice donor site of exon 3. Red dashed line magnifies pre-mRNA sequence of exon 3 and intron 3 targeted by E3I3-*sv2a* MO, uppercase letters correspond to exon 3, lowercase letters correspond to intron 3. MO targeting sequence in bold is illustrated in green brackets. (B) Blockage of E3I3 splice junction results in reduced transcript length. (C) Pilot RT-PCR confirmed that E3I3 MO (8.8 ng) was effective in disrupting correct splicing of *sv2a* pre-mRNA, resulting in abnormally spliced *sv2a* mRNA that had partial deletion of exon 3. (D) qPCR was performed to evaluate the knockdown efficiency of E3I3 MO. Increasing levels of knockdown of mature *sv2a* mRNA were observed in embryos injected with increasing amount of MO (mean±SEM). One-way ANOVA with Tukey's multiple comparison test was used. **** $p < 0.0001$. n = 3 (E) Dorsal and lateral view of a representative 6 dpf mispair CTRL MO injected larva (top) and 6 dpf E3I3 MO injected larva (bottom). Scale bar = 0.5mm. Except for the lack of swimming bladder, E3I3 morphants were indistinguishable from control MO injected larvae which displayed a normal phenotype. Moreover, there were no signs of necrosis, axis truncation, pericardial oedemas or any other dysmorphologies indicating possible off-target effects (F) Percent of animals developing epileptiform brain activity. Similar to *sv2a*$^{-/-}$ larvae (Figure S1H-I), recurrent spontaneous epileptiform events occurred in all morphants, whereas control MO injected larvae only displayed baseline activity. Significance was calculated by Fisher's exact test. **** $p < 0.0001$. n = 11-16. (G) Frequency of spontaneous epileptiform events (mean±SD) in 6 dpf E3I3 MO injected larvae was significantly higher compared to control MO injected larvae during 10 min LFP recording. Mann-Whitney test was used. **** $p < 0.0001$. n = 11-16. (H) Percent of animals developing epileptiform brain activity. Significance between 6 dpf *sv2a*$^{+/+}$ and *sv2a*$^{-/-}$ larvae was calculated by Fisher's exact test. *** $p < 0.001$. (I) Frequency of spontaneous epileptiform events (mean±SD) in 6 dpf *sv2a*$^{+/+}$ and *sv2a*$^{-/-}$ larvae during 10 min LFP recording. Mann-Whitney test was used. *** $p < 0.001$
