# Supplementary material for: Connectivity Mapping Using a Novel sv2a Loss-of-Function Zebrafish Epilepsy Model as a Powerful Strategy for Anti-epileptic Drug Discovery
Source: Front Mol Neurosci. 2022 May 24;15:881933. doi: 10.3389/fnmol.2022.881933 (PMC9172968; doi:10.3389/fnmol.2022.881933)
Supplement: Supplementary file 6 [file Image_3.pdf]

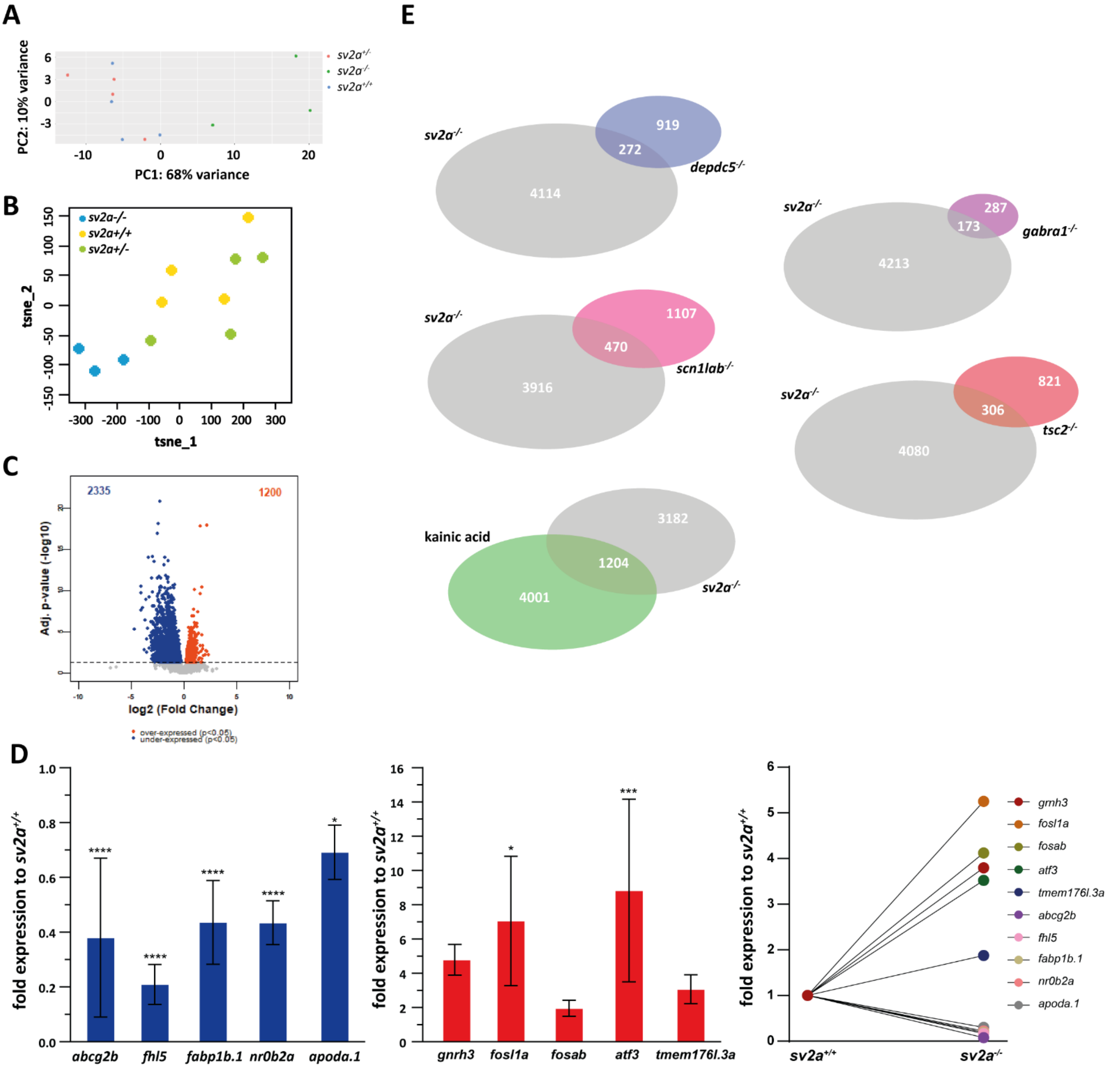

Figure S3. (A) Principal component analysis (PCA) and (B) t-distributed stochastic neighbourhood embedding (t-SNE) revealing *sv2a*$^{-/-}$ samples cluster distinctly from *sv2a*$^{+/-}$ siblings and wildtypes. (C) Volcano plot showing the DEGs (padj < 0.05) between *sv2a*$^{-/-}$ and *sv2a*$^{+/-}$. Of the 3535 DEGs, 2335 were found to be down- (blue) and 1200 to be up-regulated in *sv2a* homozygotes when compared to heterozygotes. (D) RT-qPCR validation of RNA-Seq data. Left panel demonstrating *abcg2b*, *fhl5*, *fabp1b.1*, *nr0b2a* and *apoda.1* are significantly down-regulated in *sv2a*$^{-/-}$ when compared to wildtype. Middle panel demonstrating *fosl1a* and *atf3* are significantly up-regulated in *sv2a*$^{-/-}$ when compared to wildtype, a non-significant trend was observed for *gnrh3*, *fosab* and *tmem176l_3a*. Right panel giving a visual overview of the fold changes of tested genes in RNA-Seq analysis of *sv2a*$^{-/-}$ compared to wildtype. * $p < 0.05$, *** $p < 0.001$, **** $p < 0.0001$ (E) Overlap of differentially expressed genes between *sv2a*$_{-/-}$ zebrafish larvae and *depdc5*$^{-/-}$, *gabra1*$^{-/-}$, *tsc2*$^{-/-}$, *scn1lab*$^{-/-}$ and kainic acid injected larvae.
