# Supplementary material for: Connectivity Mapping Using a Novel sv2a Loss-of-Function Zebrafish Epilepsy Model as a Powerful Strategy for Anti-epileptic Drug Discovery
Source: Front Mol Neurosci. 2022 May 24;15:881933. doi: 10.3389/fnmol.2022.881933 (PMC9172968; doi:10.3389/fnmol.2022.881933)
Supplement: Supplementary file 7 [file Image_4.PDF]

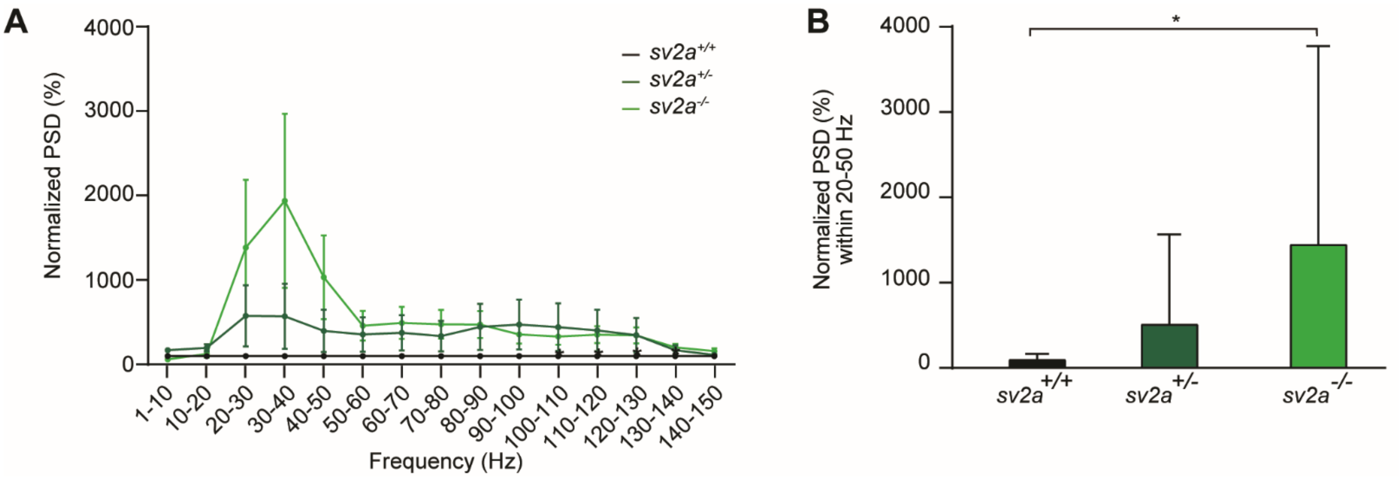

Figure S4. Spontaneous electrographic seizures in *sv2a* knockout zebrafish at 5 dpf. (A) Power spectral density (PSD) analysis of *sv2a*$^{+/+}$ (n=14), *sv2a*$^{+/-}$ (n=10), and *sv2a* $^{-/-}$ (n=9) zebrafish larvae at 5 dpf. An increase in PSD values was observed in *sv2a* $^{-/-}$ larvae compared to *sv2a*$^{+/+}$ larvae. Results were normalized to *sv2a*$^{+/+}$ larvae as 100%. (B) PSD values (mean±SD) plotted per condition over the 20–50 Hz region. Statistical analysis was performed using one-way Anova followed by Dunnett's multiple comparison (*$p < 0.05$).
